# Why aren’t we doing better in asthma: time for personalised medicine?

**DOI:** 10.1038/npjpcrm.2015.4

**Published:** 2015-03-05

**Authors:** Mike Thomas

**Affiliations:** 1 Faculty of Medicine, Primary Care and Population Sciences, University of Southampton, Southampton, UK; 2 Univer sity Hospital Southampton NHS Foundation Trust, Southampton, UK; 3 NIHR Southampton Respiratory Biomedical Research Unit, Southampton, UK; 4 NIHR Collaboration for Leadershi p in Applied Health Research and Care, Southampton, UK

## Abstract

After decades of improvement, asthma outcomes have stalled. Mortality, hospitalisations, exacerbations and symptom control remain sub-optimal. In controlled trials, most patients gain high levels of control, but in ‘real-life’ routine clinical practice most patients do not. Avoidable factors are found in most asthma deaths and hospital admissions. This perspective paper considers and contextualises the factors underlying poor asthma outcomes, and it suggests approaches that could improve the situation. Factors discussed include severe, therapy-resistant disease and the role of new and upcoming pharmacological therapies in improving outcomes. These are likely to be beneficial when targeted on patients with severe disease and discrete phenotypic characteristics, identified through biomarkers. However, for the majority of patients treated in the community, they are unlikely to be used widely, and better use of current therapy classes will be more important. Non-adherence with regular inhaled corticosteroid treatment and over-use of rescue bronchodilators are common, and many patients have poor inhaler technique. Self-management is frequently poor, particularly in those with psychosocial disadvantages and co-morbidities. Communication between clinicians and patients is sometimes poor, with failure to detect avoidable poor control and non-adherence, and failure to provide the necessary information and education to support efficient self-management. Strategies for improving monitoring and clinician–patient interactions to allow personalised treatment are considered. These strategies have the potential to allow individual patient needs to be recognised and efficient targeting of the variety of effective pharmacological and non-pharmacological interventions that we possess, which has the potential to improve both individual and population outcomes.

## Introduction

Despite effective therapies and an ongoing major investment on the part of commissioners of services and of research, asthma outcomes remain much worse in ‘real life’ than they could be, and much worse than those achieved in controlled trials. This perspectives paper aims to explore the complex web of factors relating to pathology, patients, clinicians and health systems to understand why we have stopped making progress and what we can do to improve outcomes.

## Improvements in asthma outcomes have stalled

The remarkable improvements in asthma outcomes at the end of the last century are among the great achievements of medicine. Despite increasing prevalence,^1^ progressive improvements occurred in hospitalisation, mortality, symptom control and quality of life, through safe, effective medication and structured, proactive care.^2^ Primary care generalists took on diagnosis and management for most patients, with hospital-based specialist care reserved for those with severe, therapy-resistant disease and poor control. In hindsight, a misplaced sense of complacency arose.

Unfortunately, the new millennium has shown that such an optimism was premature; asthma remains common, incurable and outcomes have stalled.^3^ The disease burden remains huge, resulting in similar levels of global health impairment as chronic liver disease and schizophrenia.^4^ People continue to die and experience severe, life-threatening attacks, which are frequently avoidable.^5^ Most of them experience regular symptoms^6^ and impaired ability to lead a full, productive life, resulting in large direct and even larger indirect costs.^7^ The aims of management are to reduce the impact on daily life (assessed by symptoms, quality of life, health resource use and biomarkers of disease activity) and the risk of adverse events (death, hospitalisation, exacerbations and lung damage).^8^ Surveys have shown little or no recent improvements in these outcomes in most developed countries. For instance, mortality and hospitalisation rates in the UK have not improved,^9,10^ with marked regional variations.^11^ The majority of European patients continue to report significant symptoms,^6^ except countries prioritising asthma as a public health problem.^12^ This has occurred despite a continual stream of new licensed asthma products, and evidence that in tightly structured clinical trial settings most patients can achieve good control.^13^

## Reasons for poor control: clinician-related factors

Although a huge effort has gone into producing and updating asthma guidelines aimed largely at primary care practitioners, clinician adherence with, and implementation of, guidelines is frequently poor. Both diagnostic and prescribing patterns often do not accord with actual care.^9^ For instance, guidelines recommend that quality-assured spirometry should be performed on all patients with suspected asthma to demonstrate obstruction and reversibility; yet, objective lung function is often not measured at all, and there are ongoing issues about the consistency of the quality of primary care spirometry.^14^ In cases of diagnostic uncertainty, guidelines recommend the use of objective tests such as measurement of bronchial hyper-reactivity or airways inflammation, but in practice these are usually not currently available to primary care clinicians in most health systems. Audits of prescribing patterns in primary care clinicians often show prescribing not in accord with guideline recommendations,^15^ including overprescribing of rescue bronchodilators, underuse of inhaled corticosteroids (ICS), use of long-acting β_2_-agonists (LABAs) as monotherapy and over-use of high doses of inhaled ICS, particularly in children.^16^ These observations point to an ongoing problem of dissemination of best practice and a likely unawareness of guidelines and of spirometry performance or interpretation in some practitioners, with a need for better education and implementation strategies. It is also clear that many patients find it hard to engage with the systems of health care that have been developed for asthma and other long-term conditions that we are unable to cure. Although best care for chronic diseases is proactive and involves self-management education, many patients only attend for care when in a crisis. Increasing numbers of patients have one or more long-term conditions (multimorbidity), yet systems of care are usually directed towards single conditions.^17^

## Reasons for poor control: therapy-resistant disease

The ‘stepped’ pharmacotherapy approach of asthma guidelines perhaps encourages the belief that ‘stronger’ medication is needed when the control is poor. Undoubtedly, some patients have intrinsically severe, therapy-resistant disease, requiring more effective treatments. However, even in this group, good quality management (including effective education in self-management) can often improve outcomes.^18^ The complexity and heterogeneity of asthma is increasingly understood, particularly in difficult asthma, and the phenotypes^19^ and endotypes^20^ constituting the asthma syndrome are better described, allowing specific groups to be identified and targeted. However, severe asthma only affects 5–10%,^9^ and even here behavioural factors are common.^21^ Greater understanding of biological abnormities with the use of biomarkers to define ‘responder’ groups allows targeting of expensive treatments involving monoclonal antibodies. Anti-IgE is now a part of care, and other mediator modulators are in late-phase clinical trials.^3^ We can be optimistic that new treatments will soon be available and, if used appropriately in dedicated ‘difficult asthma’ clinics with the necessary expertise, will be clinically effective, as well as cost effective. Indiscriminate use in everyone doing badly will not work. Psychosocial problems and non-adherence are particularly common in this group, and the involvement of primary care in coordinated, integrated multi-disciplinary care is vital.^22^

Yet majority of patients rarely see a hospital pulmonologist, and the control remains poor in those with ‘milder’ disease. Although some may have severe disease, the likelihood is that for most of them the reasons lie elsewhere. The UK National Review of Asthma Deaths has shown that most deaths occurred in those considered ‘mild’.^5^ Few advances have occured in pharmacotherapy since the leukotriene receptor antagonists, which have a role as an add-on therapy. The mainstay of treatment remains ICS, with LABA as first choice add-on therapy. Newer ICS and LABA molecules show some advantages in pharmacological properties, which may result in limited incremental clinical improvements for some; however, benefits largely relate to medication classes. Once-daily dosing may improve adherence and is preferred by patients, although the benefits over twice-daily regimes are not clear. The variety of inhaler delivery devices grows, with some being easier to use and to teach. However, despite the profusion of devices and molecules, control remains far from optimal.

## Patient-related factors in poor control: adherence and inhaler technique

Possibly the most common reasons for poor control are that many patients either do not take treatment regularly or have poor inhaler technique. Asthma is characterised by persistent airways inflammation, although manifestations are at times subclinical. The evidence for ICS is for regular use, and non-adherent patients do not get into trials or are excluded as protocol violators. However, in ‘real life’, adherence problems are ubiquitous. Regular ICS protect against death,^23^ hospitalisation,^24^ exacerbations^25^ and improve symptom control and quality of life. It is a source of bewilderment to clinicians that many patients use ICS sporadically or not at all. Adherence rates in asthma range from 30 to 70%, with under 50% of children adhering to prescribed regimens.^26^ Non-adherence with ICS is associated with progressively worse outcomes as use decreases,^19,20^ and it is common in asthma deaths.^5^ It is estimated that each 25% increase in time without ICS medication results in a doubling of the rate of asthma-related hospitalisations.^27^ The reasons for non-adherence are complex, and they include ‘non-intentional’ non-adherence—for example, forgetting medication—and ‘intentional’ non-adherence—a conscious decision not to use medication as prescribed. From their perspective, patients make a rational decision, based on their assessment of health needs and risks. Many overestimate the ICS-related risks and underappreciate the benefits of regular treatment. Some worry that the ICS ‘wear off’ with regular use despite the lack of objective evidence of tachyphylaxis. The relatively slow time course of ICS leads some to conclude that they do not work for them, particularly in comparison with the rapidly perceptible effects of bronchodilators. As many patients experience asthma as being intermittent, some only recommence ICS when symptomatic. LABAs are an effective add-on therapy, but they are unlicensed and associated with the risk of adverse events and even death when used without ICS.^28^ Unfortunately, this pattern of use continues to occur in real life, sometimes with tragic results.^5^

We encourage self-management, which is clearly necessary in a variable long-term condition. However, we fail to appreciate that non-adherence is also a form of self-management, although based on misunderstandings and inadequate information, and thus it is fundamentally a problem of inadequate patient–clinician communication. Some clinicians ‘blame’ patients for non-adherence, considering it irresponsible, a breach of the doctor–patient relationship and beyond their remit to remedy;^29^ yet, it is likely that adherence would increase if the rationale was explained in a way they understood and assimilated. Self-management education,^30^ involving patients in decisions,^31^ simplified treatment regimes^32^ and discussion of non-adherence when detected (for example, through the monitoring of refill prescription rates)^33^ all improve adherence and outcomes. There is a growing interest in using IT to support self-management and education.^34^ To progress, we have to work in partnership with patients, respecting their autonomy and recognising that they will self-manage most of the time whatever we do. Our responsibility is to ensure that their decisions are based on good information.

Correct inhaler technique is necessary for medication delivery and again the guideline evidence comes from fully trained patients demonstrating good technique. Unfortunately, in ‘real life’, many patients use their inhaler badly—most will make some errors and many make ‘critical errors’ that result in little or no medication delivery. It is unsurprising that there is a relationship between the number of errors and asthma control.^35^ Some inhalers are easier than others, and often the least expensive devices are hardest to use and teach.^36^ Ideally, all would be educated in inhaler technique by suitably qualified professionals when first prescribed, and checked at least annually and after loss of control. However, once again in ‘real life’ this does not happen frequently—some are never instructed, other than from printed packaging inserts, and many are not periodically reassessed and corrected.^37^ Indeed, some clinicians cannot demonstrate the correct technique themselves. It is incumbent on the prescriber to ensure correct technique, either personally or delegating to colleagues—for example, nurses or pharmacists.

## Patient-related factors in poor control: psychosocial and behavioural factors

As an incurable, life-long condition, asthma heavily affects well-being. Friends, colleagues and sometimes health professionals can fail to appreciate fully the impact asthma has on people’s lives. The recurrent and unpredictable experience of having to struggle to breathe is frightening and disturbing, and it can undermine well-being and stability. Those with other life stressors, such as co-morbidity, psychosocial disadvantage or with genetically or environmentally programmed lack of resilience, may experience symptoms as more distressing. All clinicians know that some patients cope better with illness than others. Some do well despite objectively severe asthma, whereas others have high symptom levels and multiple subjective problems despite apparently mild disease. Patient-reported outcomes, such as symptoms and quality of life, correlate poorly with ‘objective’ physiological and pathological parameters of disease control,^38,39^ although they are strongly correlated with psychosocial measures.^40^ Socioeconomic factors, multimorbidities and psychological state^41^ have a strong relationship with asthma outcomes of every type, and anxiety is the strongest independent predictor of the unpleasantness of breathlessness for a given degree of brochoconstriction.^42^ Symptoms, quality of life, health resource use, exacerbation frequency and even mortality are independently related to psychological state, with one study reporting psychiatric co-morbidity accounting for 29% of symptom variance.^43^

Psychological dysfunction is six times as common in people with asthma,^37^ and asthma-related quality of life correlates more closely with psychological and social status than lung function or treatment step.^44^ The underlying mechanisms probably relate to a variety of overlapping biological and behavioural factors. Functional neuroimaging has shown that brain structures mediating breathlessness are closely related anatomically and functionally to those processing emotions,^45^ and emotional state may influence immunological responses.^46^ Anxiety is associated with impaired self-management.^47^ Hyperventilation and dysfunctional breathing are associated with anxiety, occur in asthma and may trigger bronchoconstriction and asthma-like symptoms,^48^ with simple breathing control exercises improving symptoms and quality of life.^49^ A certain amount of anxiety is natural and inevitable with distressing symptoms, such as dyspnoea, and it can promote appropriate responses such as seeking help or using necessary medication, but excessive anxiety or inappropriate responses can result in a ‘negative feedback’ situation of worsening symptoms leading to further emotional and cognitive distress.

## Moving forward: how can we do better?

We need to get smarter in asthma. We do need new treatments, but we can do much better with what we already have. We require appropriately trained primary care professionals who are adequately resourced to provide quality asthma care. The first requirement is to detect poor control and risk. Unfortunately, patients may not volunteer how symptomatic they are, having grown accustomed to this over the years, and clinicians may not ask probing-enough questions. Structured reviews incorporating objective symptom assessment (ideally using validated questionnaires) will often uncover unmet need. Risk stratification to identify those at higher risk of attacks is feasible and can lead to better outcomes.^50^ Assessing rescue bronchodilator use (for example, by repeat prescription monitoring) is often revealing. Having uncovered poor control and risk, the next step is to understand why. Stepping up pharmacotherapy may be right for some patients, but addressing adherence, technique, rectifying self-management deficiencies and identifying co-morbidities (for example, anxiety, rhinitis, obesity, smoking) may be helpful in others. Simple behavioural techniques such as breathing exercises or anxiety management may help appropriately identified patients. For those needing increased treatment, better characterisation may lead to more rational and targeted treatment—for example, biomarker evidence of ongoing inflammation may point to the need for more effective anti-inflammatory treatment rather than increased bronchodilatation.^51^ Current guidelines provide a stepped approach for all patients as if they were similar, based on group mean data from clinical trials. However, responder analyses reveal remarkable heterogeneity of response to different treatments.^52^ The future lies in a stratified, individualised approach to asthma care;^53^ this is starting to occur at the severe end of the spectrum, but may be equally feasible and cost effective in wider asthma populations. We have a range of assessment options, including monitoring medication use, validated patient-reported outcome measures and simple, near-patient objective tests and a variety of treatment options (both pharmacological and non-pharmacological) that we need to target appropriately. Having spent years dissecting asthma and unravelling its complexity, we now need to put this information together into coherent, patient-orientated personalised care.

## Conclusion

Although asthma outcomes have stalled, we should use this as a spur to changing models of care rather than as a source of gloom. Our ever-increasing knowledge of the complexity and heterogeneity of asthma, together with a better understanding of how patient and clinician behaviour can be modified to make better use of the resources we do possess, allows us potential new avenues in care. It is unlikely in the foreseeable future that we can ‘cure’ asthma, but we should be able to characterise our patients better, in particular those who, for one reason or another, are not doing well, and for most there will be effective strategies for improving outcomes. However, the most effective intervention(s) will vary greatly between patients, and a ‘one size fits all’ approach will no longer suffice. As with other long-term conditions, we need to help our patients cope with the diverse consequences of having an illness that we can control but not take away.

## Notes

Neither MT nor any member of his close family has any shares in pharmaceutical companies. In the past 3 years, he has received the speaker’s honoraria for speaking at sponsored meetings or satellite symposia at conferences from the following companies marketing respiratory and allergy products: Aerocrine, Astra Zeneca, Boehringer Inglehiem, GSK, MSD, Novartis and Teva. MT is the former chief medical adviser to the charity Asthma UK, a member of the BTS SIGN Asthma guideline group and the NICE Asthma guideline group. MT is an Associate Editor of *npj Primary Care Respiratory Medicine*, but was not involved in the editorial review of, nor the decision to publish, this article.
